# Sleep in Spouses of Patients with Obsessive–Compulsive Disorder

**DOI:** 10.3390/brainsci16101122

**Published:** 2026-10-17

**Authors:** Mehmet Celal Kefeli, Mehmet Hamamcı

**Affiliations:** 1 Department of Psychiatry, Faculty of Medicine, Erzincan Binali Yıldırım University, 24100 Erzincan, Turkey; 2 Department of Neurology, Faculty of Medicine, Düzce University, 81620 Düzce, Turkey; drmehmetmehmet@gmail.com

**Keywords:** sleep quality, sleepiness, insomnia, sleep disorders, partner, spouses of psychiatric patients

## Abstract

**Highlights:**

**What are the main findings?**

- Spouses of individuals with obsessive–compulsive disorder (OCD) experience significantly poorer overall sleep quality and increased daytime sleepiness compared to healthy controls.
- Female spouses of OCD patients are disproportionately affected, exhibiting significantly higher levels of insomnia severity and sleep disturbances than their male counterparts.

**What are the implications of the main findings?**

- Psychiatric evaluations of OCD may benefit from extending beyond the patient to routinely screen partners for sleep disturbances and psychological distress, recognizing them as a high-risk demographic.
- Clinicians could consider addressing support and interventions for spouses to mitigate the secondary health impacts of the disorder and improve overall family well-being.

**Abstract:**

Background/Objectives: Obsessive–compulsive disorder (OCD) severely impacts both patients and their families. This study aimed to compare sleep quality, insomnia severity, and daytime sleepiness in partners of individuals with OCD against a healthy control group. Additionally, it investigated how patient OCD severity and partner depression and anxiety symptoms relate to these sleep outcomes. Methods: This cross-sectional case–control study included 104 spouses of OCD patients (46 females, 58 males; mean age 37.92 ± 8.22 years) and 100 healthy control spouses (48 females, 52 males; mean age 38.40 ± 7.93 years), all married for at least one year. Spouses completed the Beck Anxiety Inventory (BAI), Beck Depression Inventory (BDI), Pittsburgh Sleep Quality Index (PSQI), Epworth Sleepiness Scale (ESS), and Insomnia Severity Index (ISI). Patient OCD severity was assessed using the Yale-Brown Obsessive Compulsive Scale (Y-BOCS). Results: OCD spouses exhibited significantly higher anxiety and depression scores than controls. Poor sleep quality was reported by 57.7% of OCD spouses versus 32% of controls. After adjusting for confounders (age, body mass index, education, sex, marriage duration), OCD spouses demonstrated significantly worse sleep quality and higher daytime sleepiness. Female spouses experienced a significantly more severe burden of insomnia and sleep disturbances than males. Independent of group, higher depression scores correlated with poorer sleep parameters. Furthermore, patient OCD severity positively correlated with all partner sleep scale scores. Conclusions: We conclude that partners of individuals with OCD are particularly vulnerable to complex sleep difficulties, including poor sleep quality and daytime fatigue. By broadening our clinical focus to address these hidden sleep issues and psychological needs, we may help mitigate secondary health risks and improve the overall well-being of affected families.

## 1. Introduction

Obsessive–compulsive disorder (OCD) affects 1–3% of the general population; it is relatively more common in women and typically emerges during adolescence or late adulthood [1,2]. The disorder is characterized by recurrent and intrusive thoughts, images, or urges that cause significant distress, alongside repetitive behaviors or mental acts performed to alleviate such distress [3]. Compared to healthy people in control group, patients with OCD exhibit impaired functioning and a lower quality of life, particularly in occupational, social, and family domains [4,5,6]. It has been reported that the quality of life in individuals with OCD is equivalent to that of patients with schizophrenia while being notably worse than that observed in individuals with heroin dependence and depressive disorders [7].

OCD is a condition that impairs an individual’s functionality and quality of life, while also adversely affecting the quality of life of those cohabiting with them [8]. The rates of divorce and relationship dissolution among individuals suffering from this disorder are significantly higher compared to the general population [9]. A multitude of factors underlie this situation, categorized as the direct influence of obsessive–compulsive symptoms, specific non-symptomatic characteristics of individuals with OCD manifested within the relationship, and “partner accommodation”—the accommodating behaviors exhibited by partners in response to the patient’s symptoms. Partner accommodation is defined as a process characterized by the involvement or assistance of the spouse and other family members in the rituals of the individual with OCD [10,11]. More than 95% of families of patients with OCD assist in trigger avoidance, provide reassurance, participate in compulsive behaviors, wait for the completion of rituals, or comply with the patient’s rituals [12,13,14]. Research conducted in Japan indicated that over 40% of individuals with OCD reported family involvement or assistance in their compulsive rituals [15]. Similarly, studies conducted in Turkey have shown that family accommodation is extremely prevalent among relatives of individuals with OCD, with over 90% engaging in accommodation behaviors and nearly 38% exhibiting these supportive or accommodating behaviors on a daily basis [16]. This phenomenon compels family members, particularly spouses, to adopt behavioral patterns akin to those of the OCD patients themselves. From this perspective, families of patients with OCD are distinctively characterized by a more pronounced stressor load compared to families of individuals with other mental disorders. Beyond this, it is common for the family members of OCD patients to take over the patient’s daily tasks and functional responsibilities [17]. This situation constitutes another significant stressor for the spouses of patients with OCD. For this reason, an increasing number of studies have recently centered their focus beyond OCD patients to include the quality of life, coping strategies, and other psychological profiles of their family members [8,18,19,20]. Recent studies on families dealing with OCD highlight a clear pattern: relatives frequently face high levels of stress and unhelpful cognitive styles. We see these elements feeding into a broader ecosystem of shared psychological distress within the household [21]. Multiple investigations [22,23] have documented a positive correlation between sleep disorders and quality of life. In this context, it is highly probable that spouses of individuals with OCD exhibit a higher frequency of sleep disorders.

Sleep is a universal function across living species and constitutes approximately one-third of the human lifespan. Recent studies have revealed that sleep is influenced by a broad spectrum of factors, ranging from severe medical conditions such as head and neck cancers to lifestyle elements like the positive impact of the Mediterranean diet on sleep quality [24,25]. Sleep disorders are prevalent conditions that can lead to serious consequences for patients’ quality of life and overall health [26]. Sleep disorders can present either in isolation or as concomitant features of a wide range of psychiatric and neurological illnesses. Numerous studies have investigated sleep disturbances in patients with OCD, demonstrating that the prevalence of sleep disorders is significantly higher in this population compared to the public [27]. Nevertheless, as far as we are aware, no research has yet explored this condition among spouses, despite their status as the group most psychosocially impacted by OCD symptoms following the patients themselves.

When investigating sleep disturbances in the spouses of patients with OCD, the comorbidity of depression is a critical factor that must not be overlooked. Indeed, research has demonstrated that depression levels among the relatives of OCD patients are significantly higher than those in the general population [8,19]. Depression is widely recognized as a significant contributor to the development of sleep disorders. On the other hand, since sleep-related symptoms, including insomnia and nightmares, are part of the diagnostic criteria for certain anxiety disorders, investigating anxiety as a comorbid factor becomes imperative [28].

The primary aim of this study was to compare and evaluate sleep quality, insomnia severity, and daytime sleepiness in the partners of individuals with obsessive–compulsive disorder (OCD) to a healthy control group. Additionally, as a secondary aim, this study aimed to investigate the relationship between patient-related OCD severity, partner-related depression and anxiety symptoms, and sleep-related outcomes.

## 2. Materials and Methods

### 2.1. Study Population

This cross-sectional case–control study was conducted at a tertiary hospital between 15 June 2025, and 15 December 2025. The study was carried out in accordance with the principles of the Declaration of Helsinki, and written informed consent was obtained from all participants. While the study primarily targeted the spouses, clinical data from the OCD patients were only used after obtaining their detailed written informed consent. Considering their psychiatric vulnerability, special care was taken to ensure their participation was entirely voluntary and independent of their spouses. The study received ethical approval by Institutional Review Board of Erzincan Binali Yıldırım University Faculty of Medicine (Protocol No:2025-10/01) 29 May 2025.

The study included spouses of patients aged 19–60 who had been married for at least one year and were diagnosed with OCD by experienced psychiatrists according to DSM-5 criteria [3]. The healthy control group comprised spouses who had been married for a minimum of one year, recruited from hospital staff, non-psychiatric outpatients’ accompanying persons, and local community volunteers. The control group was frequency-matched (group-level matching) to the OCD spouse group based on age and sex distributions. The healthy status and absence of any psychiatric disorders were verified through detailed clinical interviews and mental status examinations conducted by experienced psychiatrists for both the control participants and their respective partners, alongside the strict application of the study’s exclusion criteria. The sample size of this study was determined based on a time-anchored, consecutive sampling approach within a predefined six-month enrollment period (between 15 June 2025 and 15 December 2025). During this timeframe, all consecutive eligible participants who met the inclusion criteria and agreed to participate were recruited. Initially, a total of 163 married couples, in which one partner was diagnosed with OCD, were evaluated for the study. Following the strict application of inclusion and exclusion criteria during this specific study period, 104 spouses of OCD patients were enrolled in the final analysis. For the control group, 100 spouses of healthy individuals with no history of psychiatric disorders were recruited using the same consecutive enrollment strategy within the identical timeframe. A subsequent post hoc power analysis confirmed that this final sample size provided sufficient statistical power (84.1%) for the main analyses, based on an effect size of 0.38 and a significance level of α = 0.05.

Volunteers with at least a primary education level and the cognitive capacity to understand the study’s scope and complete the questionnaires were included in the research. Individuals with alcohol or caffeine dependence, chronic physical illnesses, shift workers, pregnant or lactating women, smokers, patients with diagnosed psychiatric or neurological disorders, and those with endocrine or systemic diseases were excluded from the study. The control group consisted of 100 healthy volunteers—matched for age and sex with the OCD spouse group—whose healthy status and absence of any psychiatric disorders were verified through detailed clinical interviews and mental status examinations conducted by experienced psychiatrists, alongside the strict application of the study’s exclusion criteria.

### 2.2. Evaluation Tools

Initially, a detailed clinical history was obtained from the patients with OCD to ensure they had no other chronic illnesses before their spouses were enrolled in the study. A systemic physical examination and a mental status examination were conducted. Eligible OCD patients were assessed using the Yale-Brown obsessive–compulsive Scale (Y-BOCS).

A detailed clinical history was obtained from the participants. Systemic physical and mental status examinations were conducted. Height and weight were measured, and the Body Mass Index (BMI) was calculated and recorded. All participants were administered a Data Collection Form, the Beck Anxiety Inventory (BAI), the Beck Depression Inventory (BDI), the Pittsburgh Sleep Quality Index (PSQI), the Epworth Sleepiness Scale (ESS), and the Insomnia Severity Index (ISI).

- Data Collection Form: Developed by the researchers for the purposes of this study, included items regarding the participants’ life histories and general sociodemographic information.
- Beck Anxiety Inventory (BAI): Developed by Beck et al. [29] to measure the severity of an individual’s anxiety symptoms. The Turkish validity and reliability study of the scale was conducted by Ulusoy et al. [30].
- Beck Depression Inventory (BDI): Developed by Beck et al. [31] to evaluate and rate the somatic, emotional, and motivational symptoms associated with depression. The Turkish validity and reliability study was conducted by Hisli et al. [32].
- Pittsburgh Sleep Quality Index (PSQI): Developed by Buysse et al. [33]. Higher scores obtained from the scale indicate poorer sleep quality. A total score of 5 or higher is indicative of poor sleep quality. The Turkish validity and reliability study of the scale was conducted by Ağargün et al. [34].
- Epworth Sleepiness Scale (ESS): Developed by M.W. Johns in 1991 [35]. It is utilized to assess daytime sleepiness. A total score of 10 or higher indicates excessive daytime sleepiness. The Turkish validity and reliability study of the scale was conducted by Izci et al. [36].
- Insomnia Severity Index (ISI): Developed by Bastien et al. [37] to screen for and assess the severity of insomnia symptoms. The Turkish validity and reliability study of the index was conducted by Boysan et al. [38].
- Yale-Brown ObsessiveCompulsive Scale (Y-BOCS): Developed by Goodman et al. [39] to measure the type and severity of obsessive–compulsive symptoms. Additionally, a symptom checklist is utilized alongside the scale to investigate the distribution of symptoms [40].

### 2.3. Statistical Analysis

Statistical analyses were performed using the SPSS^®^ version 22.0 software package (IBM Corp., Armonk, NY, USA). The conformity of the data to a normal distribution was assessed using the Kolmogorov–Smirnov test. A one-tailed post hoc power analysis was conducted using the G*Power software (v3.1.9.7) based on the PSQI total score as the primary outcome variable. With an effect size of 0.38 (derived from the Mann–Whitney U test), a significance level of α = 0.05, and a total sample size of 204 (Control = 100, S-OCD = 104), the statistical power of the study was calculated to be 84.1% (Power (1−β) = 0.84).

In descriptive statistics, categorical variables were presented as numbers (*n*) and percentages (%). Continuous variables were expressed as Mean ± Standard Deviation for normally distributed data, while non-normally distributed variables were reported as Median (25th–75th percentiles). The Chi-square test was utilized for the comparison of categorical variables. To compare the two groups (spouses of OCD patients and the control group), the Independent Samples *t*-test was performed for normally distributed data, whereas the Mann–Whitney U test was used for non-normally distributed data. Pearson or Spearman correlation analyses were performed to investigate the relationships between OCD severity (Y-BOCS) in the patient group and the scale scores of the spouses (BDI, BAI, PSQI, ISI, ESS), depending on the distribution characteristics of the data.

A multivariate analysis of covariance (MANCOVA) was initially planned to evaluate the PSQI, ESS, and ISI scores collectively as dependent variables. However, prior to the analysis, the underlying model assumptions were rigorously assessed. Due to the violation of these assumptions, Generalized Linear Models (GLM) were employed as a more flexible modeling approach to analyze each sleep parameter (PSQI total score, ISI, and ESS) separately. These models utilized a linear distribution and an identity link function. Furthermore, to mitigate potential bias arising from the inequality of variances, heteroscedasticity-consistent standard errors were preferred.

In the analyses, group (S-OCD spouses vs. controls) was defined as a fixed factor, while age, sex, BMI, education level, duration of marriage, and scores from the BDI and BAI were included as covariates accounting for concurrent psychological distress and demographic characteristics. Furthermore, two-way interaction terms between the group and each covariate were incorporated into the models to test whether demographic factors (such as sex and marriage duration) moderate the association between group membership and sleep disturbances. In the GLM analyses, Group was coded as 0 for spouses of OCD patients and 1 for healthy controls. Sex was coded as 0 for females and 1 for males. For intergroup comparisons, Estimated Marginal Means (EMM)—adjusted for the effects of covariates—and parameter estimates were reported. Statistical significance was set at *p* < 0.05 for all tests.

## 3. Results

The spouses of OCD patients and the control group were found to be similar in terms of age and sex. No significant differences were detected between the two groups regarding educational level and Body Mass Index (BMI) and duration of marriage. The OCD patient group had a mean Y-BOCS score of 22.60 ± 5.89. Sociodemographic data are presented in Table 1.

The anxiety and depression scores of the spouses of patients with OCD were statistically significantly higher than those of the control group. The ESS and ISI scores of the spouses of patients with OCD were statistically significantly higher than those of the control group. Table 2 presents the BAI, BDI, ESS, and ISI scores for both groups.

According to the PSQI scores, 32% of the control group and 57.7% of the spouses of patients with OCD had poor sleep quality. The scores of the spouses of patients with OCD were significantly higher across the subscales of sleep disturbances, daytime dysfunction, subjective sleep quality, sleep latency, sleep duration, and sleep efficiency. However, no significant difference was observed between the two groups in terms of sleep medication use (Table 3).

When sleep parameters (PSQI, ISI, and ESS) were evaluated by within-group sex comparisons (as presented in Table 4), no statistically significant differences were observed between female and male participants in the healthy control group (*p* > 0.05). However, significant sex-based differences emerged within the S-OCD group. Specifically, female spouses of OCD patients exhibited a significantly higher median total PSQI score (7 [4–9]) compared to male spouses (4 [2–9]) (U = 987.50, *p* = 0.023, r^2^ = 0.050). Similarly, ISI scores were significantly higher in female spouses of OCD patients (12.5 [8–16]) than in their male counterparts (6.5 [3.25–15]) (U = 951.00, *p* = 0.012, r^2^ = 0.060). Regarding daytime sleepiness, although female spouses in the OCD group showed slightly higher median ESS scores (7 [3–9]) than male spouses (4.5 [3–8]), this difference was at a borderline level of significance (U = 1042.50, *p* = 0.056, r^2^ = 0.035).

Age and BMI did not show any significant correlation with the scores of the sleep measures (PSQI, ESS, and ISI). A significant positive correlation was observed between Y-BOCS scores and the scores of the sleep scales (PSQI, ESS, and ISI). Significant positive correlations were observed between BAI and BDI scores and the scores of the sleep scales (PSQI, ESS, and ISI). BAI and BDI scores were not significantly correlated with Y-BOCS scores. Associations are summarized in Table 5.

As presented in Table 6, a GLM was constructed to examine the influence of group membership alongside covariates on PSQI levels. The model demonstrated a significant overall fit to the data, as confirmed by the Omnibus test (*p* < 0.001). A significant negative main effect was observed for sex (B = −1.836), indicating that males generally reported lower PSQI scores compared to females. However, a significant positive Group × Sex interaction was identified (B = 2.009, *p* = 0.020). Furthermore, a borderline significant interaction between Group and duration of marriage was observed (B = 0.206, *p* = 0.049).

As illustrated in Table 7, a GLM was established to evaluate the impact of group membership and various covariates on ESS levels. In contrast to the PSQI results, no significant interaction terms were identified in the ESS analysis. However, both BMI and BDI emerged as independent risk factors significantly influencing ESS levels when adjusting for other variables (*p* < 0.05). Specifically, increased BDI scores were independently associated with higher ESS levels (B = 0.397, *p* < 0.001). Similarly, BMI demonstrated a significant main effect on ESS scores (B = 0.305, *p* = 0.022).

As presented in Table 8, a GLM was constructed to analyze the influence of group membership and several covariates on ISI levels. A significant negative main effect was observed for sex (B = −2.755). However, the Group × Sex interaction was found to be positive and statistically significant (B = 3.505, *p* = 0.028). Independent of group membership, every one-unit increase in BDI scores was associated with a 0.51-unit increase in insomnia severity (B = 0.513, *p* < 0.001).

The adjusted marginal means for PSQI were 3.68 ± 0.47 for female controls, 3.85 ± 0.35 for male controls, 6.84 ± 0.55 for female S-OCD spouses, and 5.00 ± 0.43 for male S-OCD spouses. The corresponding ISI values were 8.27 ± 0.90, 9.02 ± 0.68, 10.63 ± 0.96, and 7.87 ± 0.70, respectively. Female S-OCD spouses had significantly higher adjusted PSQI (*p* = 0.005) and ISI (*p* = 0.026) scores than male S-OCD spouses, whereas no significant sex differences were observed in the control group.

Table 9 presents the group comparisons for PSQI, ESS, and ISI scores, adjusted for potential covariates. Variables including age, BMI, education level, sex, duration of marriage, and scores for depression (BDI) and anxiety (BAI) were incorporated into the model as covariates. With covariates held constant, statistically significant differences were observed between groups in terms of PSQI and ESS scores. In contrast, no significant difference was observed between the groups regarding ISI scores.

## 4. Discussion

In this study, we evaluated sleep disturbances, insomnia severity, and daytime sleepiness in spouses of individuals with OCD relative to a control group, while also examining the clinical and psychological factors shaping these outcomes. Our findings reveal a striking pattern: living with an OCD partner appears to be significantly associated with an increased vulnerability to poor sleep health—especially among female partners—independently of mood scores and sociodemographic variables.

The mean Y-BOCS score for the OCD patients whose spouses were enrolled in the study was 22.60 ± 5.89. Considering these findings, the sample group primarily consisted of the spouses of patients exhibiting moderate-to-severe OCD symptoms. It has been reported that a mere 28% of individuals with OCD seek professional assistance regarding their symptomatology [41]. It is estimated that only individuals with the most severe disability of obsessive–compulsive disorder utilize treatment services, while those with less distressing symptoms do not [42]. Therefore, this distribution of the data collected from the psychiatry clinic can be considered an expected outcome.

The association between body mass index and sleep disorders has been demonstrated in numerous studies [43,44]. In line with existing literature, our findings demonstrated that BMI is significantly associated with daytime sleepiness, regardless of group status. To account for this potential association, we evaluated the BMI of both groups; however, no statistically significant difference was identified between them. Although there are no studies in the literature investigating BMI specifically among the spouses of patients with OCD, the fact that OCD patients themselves do not differ from healthy controls in terms of BMI [45] makes our findings unsurprising.

In the present study, the BDI and BAI scores of the spouses of patients with OCD were found to be significantly higher than those of the control group. While a study involving relatives of individuals with OCD reported significantly higher depression levels, no significant differences were detected in anxiety rates [8]. In contrast to previous studies, the significantly higher anxiety levels observed in our sample may stem from certain non-symptom-related features of OCD, which are hypothesized to undermine marital harmony between the patient and the spouse. To illustrate, OCD patients have been shown to be less successful in their interpersonal relationships, including those within partner associations [18]. Studies have shown that patients with OCD are more likely to have anxious or avoidant attachment styles and tend to exhibit higher levels of jealousy [46]. Individuals with OCD manifest over-controlling patterns in their interpersonal dynamics, characterized by a persistent desire to regulate and maintain control over the relationship [47]. Partner-involved compulsions may be present in some cases of OCD, potentially leading to increased anxiety in spouses; for example, severe contamination obsessions may prevent individuals from participating in sexual relations altogether [48]. Moreover, recent studies have demonstrated that patients with OCD exhibit higher levels of alexithymia, greater sexual dysfunction, and reduced dyadic adjustment compared to healthy controls [49]. The dynamics mentioned above, which uniquely affect spouses without causing perceptible distress in other family members, might provide an explanation for the high levels of anxiety found among the partners. Furthermore, while no significant correlation was found between the severity of obsessive–compulsive symptoms measured by the Y-BOCS and anxiety or depression scores, the significantly elevated levels of anxiety and depression in spouses may be attributed to the aforementioned non-symptomatic factors that impact marital relationships. Another reason for the lack of a significant correlation may be the recruitment of patients from a psychiatry clinic; as many individuals with mild OCD symptoms do not seek professional help, our sample was predominantly concentrated on the spouses of patients exhibiting moderate-to-severe symptoms.

Another noteworthy finding in our study is that female spouses in the OCD group exhibited significantly worse sleep quality and higher insomnia levels. This suggests that being the spouse of an individual with OCD may be associated with an increased vulnerability to sleep disturbances specifically for women. Although men generally reported lower baseline scores across the entire cohort, the significant Group × Sex interaction told a different story. Female partners in the OCD group experienced a disproportionately sharp rise in sleep impairment, absorbing the heaviest toll of the condition. We also noted an exploratory Group × duration of marriage interaction for the PSQI (B = 0.206, *p* = 0.049). This points to a potential nuance: the link between group status and sleep quality might shift depending on how long couples have been married. Given its exploratory nature and borderline significance, we view this finding with appropriate caution.

Apart from medication use, all PSQI subscales, the total PSQI score, and the ISI and ESS scores showed a significant increase in the OCD spouse group compared to the healthy controls. These findings indicate that the spouses of patients with OCD experience poorer sleep quality across multiple dimensions, including insomnia, daytime sleepiness, sleep latency, sleep duration, habitual sleep efficiency, sleep disturbances, and daytime dysfunction. In terms of sleep quality, 32% of the control group were classified as poor sleepers, compared to a substantially higher proportion of 57.7% in the spouses of OCD patients. Although 32% of the control group exhibited poor sleep quality (PSQI ≥ 5), this finding aligns with large-scale community studies reporting general population prevalence rates of approximately 36%, largely attributable to modern lifestyle stressors, urban environmental factors, and the sensitive threshold of the PSQI scale [50]. This suggests that the situation may be associated with partner accommodation and the other aforementioned processes that disrupt the marital adjustment of individuals with OCD. Additionally, the fact that PSQI, ESS, and ISI scores correlate with Y-BOCS scores implies a direct association between sleep-related impairments and the clinical symptomatology of the disorder.

When evaluated based on the ESS cutoff scores, no significant difference was observed between the two groups. This finding was likely attributed to the fact that sleepiness levels rarely reached clinical significance within the groups.

While initial correlations indicated that both anxiety and depression contribute to the sleep disturbances observed, subsequent multivariate analyses revealed that anxiety was not an independent predictor of sleep outcomes. Conversely, the literature has established a clear relationship between anxiety disorders and impaired sleep quality, with evidence indicating that the risk of sleep disturbances is increased twofold in individuals with generalized anxiety disorder [51,52]. However, in line with prior research, depression emerged as a significant factor associated with diminished sleep quality, increased sleepiness, and greater insomnia, irrespective of group assignment [53,54]. As detailed in Table 5, Beck Depression Inventory scores correlated positively with sleep measures across our cohort. At the same time, we found no direct link between patient symptom severity (Y-BOCS) and partner depression scores. This divergence highlights that depressive symptoms experienced by spouses stem less from the raw severity of the patient’s obsessive–compulsive symptoms and more from broader interpersonal dynamics and partner accommodation patterns.

Studies conducted among the spouses of individuals with chronic illnesses that impair quality of life, such as Parkinson’s disease and epilepsy, have observed a significant decline in sleep quality [55,56]. In instances such as nocturnal epilepsy, where the patient’s sleep cycle is compromised, it is known that spouses experience even greater sleep disturbances [55]. In patients with OCD, for whom an increased prevalence of insomnia has been demonstrated in numerous studies, it is suggested that the potential interference of persistent thoughts and compulsions with sleep may be a contributing factor. Overall, these studies have demonstrated issues such as insomnia, poor sleep quality, and difficulties in initiating and maintaining sleep [25]. The increased prevalence of sleep disorders in patients with OCD, combined with the observation that sleep disturbances are more common among the spouses of individuals with such issues, may represent an additional factor in the development of sleep disorders in the spouses of patients with OCD [26,57,58].

Certain limitations of this study warrant consideration. First, as the data were gathered from a tertiary care setting, the sample predominantly featured moderate-to-severe OCD cases. Consequently, our inability to recruit enough spouses of patients with mild symptoms may limit the generalizability of the results across the entire clinical spectrum. A further shortcoming of the present study pertains to the use of subjective scales instead of objective assessment tools, such as wrist actigraphy monitoring or polysomnography, for gathering sleep data—a factor that future research should aim to rectify. Our cross-sectional design required controlling for psychological distress metrics, including BDI and BAI, as covariates. Yet, this introduces a analytical gray area. These scales might act strictly as covariates, or they could function as intermediate mediators along the causal pathway linking partner OCD exposure to sleep disruption. We cannot definitively tease apart these dual roles with cross-sectional data alone.

## 5. Conclusions

In conclusion, our findings highlight that spouses of individuals with obsessive–compulsive disorder experience complex sleep disturbances compared to a healthy control group. In analyses adjusted for age, BMI, education level, sex, marriage duration, and scores of depression (BDI) and anxiety (BAI), it was determined that higher daytime sleepiness scores and sleep quality impairment were observed among the spouses of individuals with OCD. The findings suggest that spouses of individuals with OCD appear to represent a population vulnerable to sleep disturbances. The current literature suggests that, through spousal adaptation and related mechanisms, relatives may become so focused on the patient and clinical symptoms that they neglect their own psychological needs [59]. The findings of our study may improve the quality of social life by shifting clinicians’ focus to the patients’ relatives, thus helping to recognize and remain mindful of often unaddressed sleep disturbances. We believe that incorporating partner-focused assessments into routine psychiatric care could help clinicians recognize and address family-wide distress. This is the first study to address this issue; however, further research is needed to confirm these findings and conduct a more comprehensive assessment of the underlying pathophysiology.

## Figures and Tables

**Table 1:** Demographic characteristics of the spouses of patients with obsessive–compulsive disorder and control group.

Variable	Control (n = 100)	S-OCD (n = 104)	Test Statistic	*p*-Value
Age	38.40 ± 7.93	37.92 ± 8.22	t(202) = 0.421	0.674
Sex (Female/Male)	48 (48%)/52 (52%)	46 (44.2%)/58 (55.8%)	χ^2^(1) = 0.292	0.589
Duration of marriage, years	9.5 (4–17)	8 (2–14)	U = 4636.50	0.177
Education level	11 (8–15)	11 (8–13)	U = 4633.50	0.174
BMI	25.29 ± 3.42	26.08 ± 4.15	t(202) = 1.478	0.141
YBOCS	--	22.60 ± 5.89	--	--

Data were presented as n (%) or mean ± (SD) or median (Q1–Q3).

**Table 2:** Comparison of the two groups according to Beck Depression inventory, Beck Anxiety inventory, Epworth Sleepiness scale and Insomnia Severity index scores.

Variable	Control (n = 100)	S-OCD (n = 104)	Test Statistic	*p*-Value	Effect Size
BDI median (Q1–Q3)	6 (4–9)	9 (5–14)	U = 3507.50	<0.001	r^2^ = 0.079
BAI median (Q1–Q3)	6 (4–8)	8 (4–14)	U = 3556.00	<0.001	r^2^ = 0.075
ESS median (Q1–Q3)	3 (0–5)	6 (3–9)	U = 3072.50	<0.001	r^2^ = 0.126
ESS ≥ 10% (n)	8 (8)	14 (13.5)	χ^2^(1) = 1.581	0.209	—
ISI median (Q1–Q3)	7 (4–11)	9 (4–15)	U = 4204.50	0.018	r^2^ = 0.027

**Table 3:** Comparison of Pittsburg Sleep Quality index total scores and subscale scores of two groups.

Variable	Control (n = 100)	S-OCD (n = 104)	Test Statistic	*p*-Value	Effect Size
PSQI total	3 (1–6)	6 (3.5–9)	U = 2851.50	<0.001	r^2^ = 0.154
Subjective Sleep Quality	1 (0–1)	1 (1–2)	U = 3724.50	<0.001	r^2^ = 0.069
Sleep latency	1 (0–1)	1 (1–2)	U = 3235.00	<0.001	r^2^ = 0.118
Sleep duration	0 (0–1)	1 (0–1)	U = 4021.50	0.002	r^2^ = 0.047
Habitual sleep efficiency	0 (0–1)	1 (0–1)	U = 4064.50	0.003	r^2^ = 0.043
Sleep disturbance	1 (0–1)	1 (1–2)	U = 3133.00	<0.001	r^2^ = 0.142
Use of sleeping medications	0 (0–0)	0 (0–0)	U = 5000.50	0.106	—
Daytime Dysfunction	0 (0–1)	1 (0–1)	U = 3408.00	<0.001	r^2^ = 0.109
Poor sleep quality	32 (32%)	60 (57.7%)	χ^2^(1) = 13.591	<0.001	φ^2^ = 0.067

**Table 4:** PSQI, ISI, and ESS Scores with Within-Group Sex Comparisons.

Group	Median (Q1–Q3)	Test Statistic	*p*-Value	Effect Size (r^2^)
	PSQI total			
Healthy Control—Female (n = 48)	2 (1–6)	U = 1143.00	0.467	0.005
Healthy Control—Male (n = 52)	3 (1.75–5.25)			
S-OCD—Female (n = 46)	7 (4–9)	U = 987.50	0.023	0.050
S-OCD—Male (n = 58)	4 (2–9)			
	ISI			
Healthy Control—Female (n = 48)	6.5 (3.75–10)	U = 1160.00	0.545	0.004
Healthy Control—Male (n = 52)	8 (4–11.25)			
S-OCD—Female (n = 46)	12.5 (8–16)	U = 951.00	0.012	0.060
S-OCD—Male (n = 58)	6.5 (3.25–15)			
	ESS			
Healthy Control—Female (n = 48)	3 (0–6)	U = 1240.00	0.958	<0.001
Healthy Control—Male (n = 52)	3 (1–4)			
S-OCD—Female (n = 46)	7 (3–9)	U = 1042.50	0.056	0.035
S-OCD—Male (n = 58)	4.5 (3–8)			

**Table 5:** Relationship between ESS, ISI, and total PSQI and age, BMI, YBOCS, BDI and BAI in spouses of patients with OCD.

Variable	PSQI Total Score	ESS	ISI	YBOCS
Age	r = −0.131, *p* = 0.183	r = 0.097, *p* = 0.328	r = −0.086, *p* = 0.385	r = −0.013, *p* = 0.893
BMI	r = −0.045, *p* = 0.652	r = 0.023, *p* = 0.813	r = −0.047, *p* = 0.639	r = −0.067, *p* = 0.501
YBOCS	r = 0.530, *p* < 0.001	r = 0.367, *p* < 0.001	r = 0.395, *p* < 0.001	----
BDI	r = 0.427, *p* < 0.001	r = 0.403, *p* < 0.001	r = 0.412, *p* < 0.001	r = 0.159, *p* = 0.106
BAI	r = 0.354, *p* < 0.001	r = 0.230, *p* = 0.019	r = 0.296, *p* = 0.002	r = 0.177, *p* = 0.071

**Table 6:** Parameter estimates of selected key factors associated with PSQI scores.

Variables	B	Std. Error	Wald X^2^	*p*
Intercept	−1.883	3.299	0.326	0.568
Group	3.692	4.047	0.832	0.362
Sex	−1.836	0.657	7.822	0.005
BDI	0.258	0.066	15.323	<0.001
Group × Sex	2.009	0.862	5.430	0.020
Group × Duration of marriage	0.206	0.105	3.875	0.049

(Omnibus test: X^2^ = 84.295, *p* < 0.001).

**Table 7:** Parameter estimates of selected key factors associated with ESS scores.

Variables	B	Std. Error	Wald X^2^	*p*
Intercept	−5.898	4.141	2.029	0.154
BMI	0.305	0.133	5.242	0.022
BDI	0.397	0.083	22.714	<0.001

(Omnibus test: X^2^ = 80.246, *p* < 0.001).

**Table 8:** Parameter estimates of selected key factors associated with ISI scores.

Variables	B	Std. Error	Wald X^2^	*p*
Sex	−2.755	1.235	4.974	0.026
BDI	0.513	0.121	17.897	<0.001
Group ×Sex	3.505	1.598	4.808	0.028

(Omnibus test: X^2^ = 56.979, *p* < 0.001).

**Table 9:** Group Comparison of PSQI, ESS, and ISI Scores Based on Adjusted Means.

Dependent Variable	Healthy Control (n = 100)	S-OCD (n = 104)	Mean Difference	Wald X^2^	*p*
PSQI	3.77 ± 0.30	5.85 ± 0.37	−2.08	19.52	<0.001
ESS	4.00 ± 0.35	6.16 ± 0.44	−2.15	14.50	<0.001
ISI	8.67 ± 0.60	9.14 ± 0.56	−0.47	0.33	0.565

Data are presented as adjusted mean ± standard error (SE).

## Data Availability

The datasets generated and/or analyzed during the current study are not publicly available due to privacy and ethical restrictions regarding participant confidentiality, but are available from the corresponding author on reasonable request.
